# Tongue muscle strength affects posterior pharyngeal wall advancement during swallowing: A cross‐sectional study of outpatients with dysphagia

**DOI:** 10.1111/joor.13120

**Published:** 2020-11-08

**Authors:** Keigo Nagashima, Takeshi Kikutani, Taishi Miyashita, Yuri Yajima, Fumiyo Tamura

**Affiliations:** ^1^ Division of Clinical Oral Rehabilitation The Nippon Dental University Graduate School of Life Dentistry Tokyo Japan; ^2^ Division of Rehabilitation for Speech and Swallowing Disorders The Nippon Dental University Tama Oral Rehabilitation Clinic Tokyo Japan; ^3^ Division of Rehabilitation for Speech and Swallowing Disorders The Nippon Dental University Hospital Tokyo Japan

**Keywords:** dysphasia, pharyngeal motion, posterior pharyngeal wall, skeletal muscles mass index, tongue pressure

## Abstract

**Background:**

Tongue muscle strength is important for swallowing but decreases with age, in association with reduced skeletal muscle mass. However, the relationships between pharyngeal dynamics and both skeletal muscle mass and tongue muscle strength are unknown.

**Objective:**

To investigate the effect of reductions in tongue muscle strength on pharyngeal movement during swallowing in patients with dysphagia.

**Methods:**

Subjects were selected from male outpatients ≥65 years old who were examined for the main complaint of dysphagia. Patients with history of neurodegenerative disease affecting tongue movement, cerebrovascular disease or oral cancer were excluded. As a result, 82 men (mean age, 80.6 ± 6.8 years) participated. Skeletal muscle mass index (SMI) as physical parameters and maximum tongue pressure (MTP) as tongue muscles strength were measured. Status and dynamics of the pharyngeal organs, including change in posterior pharyngeal wall advancement (PPWA) when swallowing 3.0 mL of moderately thick liquid, were measured by analysing videofluoroscopic images. Simple bivariate correlation and multiple regression analysis were used to statistically analyse correlations between parameters.

**Results:**

MTP showed a significant positive correlation with SMI (*r* = .43, *P* < .01). PPWA showed a significant negative correlation with MTP (*r* = −0.30, *P* < .01), but no association with SMI.

**Conclusions:**

While tongue muscle strength is affected by skeletal muscle mass, posterior pharyngeal wall advancement is not readily affected by decreases in skeletal muscle mass. Posterior pharyngeal wall advancement may increase to compensate for swallowing function among individuals with reduced tongue muscle strength.

## 1 INTRODUCTION

The tongue plays an important role in swallowing movements. Decreases in tongue pressure, as an index of tongue muscle strength, are related to the dysphagia seen in patients with neurodegenerative diseases such as amyotrophic lateral sclerosis^1^ and Parkinson's disease,^2^ and in stroke patients.^3^ Furthermore, tongue pressure decreases with age, even in the absence of disease.^4^, ^5^, ^6^

Loss of tongue pressure is known to be associated with oral‐phase dysphagia, weakening tongue movements during mastication and affecting actions such as bolus formation and transfer of food to the pharynx.^7^ Tsujimura et al^8^ reported that hypoglossal nerve transection reduced swallowing pressure at the oropharynx. However, few reports have described relationships between reduction in tongue pressure and the state or movements of the pharyngeal organs during the pharyngeal phase of swallowing.^9^, ^10^

The tongue muscles provide the tongue muscle strength needed for swallowing function and are striated muscles of somatic origin. Tongue muscle strength are believed to be susceptible to the effects of decreases in skeletal muscle mass^6^, ^11^ and hand grip strength^12^ with age. The pharyngeal muscles, on the other hand, are striated muscles originating from the fourth branchial arch. Except during swallowing, these muscles show activity linked to exhalation.^13^ Immunohistochemical studies indicate that ageing‐related changes in pharyngeal muscles may differ from the loss of muscle mass and strength commonly seen as ageing‐related changes in skeletal muscles.^14^

Furthermore, an increase in posterior pharyngeal wall advancement may occur during the pharyngeal phase of swallowing when tongue function is reduced in patients with tongue cancer, apparently as a compensatory action by the pharyngeal constrictor muscle.^15^, ^16^ Given such findings, other factor‐related reduction in tongue muscle strength on pharyngeal movements during swallowing remain unclear.

The purpose of the present study was to clarify the effects of reductions in tongue muscle strength on pharyngeal movements during swallowing in patients with dysphagia.

## 2 METHODS

### 2.1 Participants

The target group comprised 165 patients ≥65 years old who underwent male outpatient examination during the period from September 2018 to March 2020 for the main complaint of dysphagia and were evaluated for swallowing function. Of these, the following were excluded because of the possibility of direct effects on tongue movement: 19 patients with a history of neurodegenerative disease such as amyotrophic lateral sclerosis, Parkinson's disease, progressive supranuclear palsy or spinocerebellar degeneration; 43 patients with a history of stroke; and 13 patients with a history of oral cancer. In addition, we excluded 8 patients who did not complete the prescribed diagnostic examinations because they were unable to understand or follow our instructions due to serious cognitive impairment. As a result, a final total of 82 subjects (mean age, 80.6 ± 6.8 years) was analysed. This study was approved by the ethics committee of The Nippon Dental University (approval no. NDU‐T2020‐08), and complied with the principles of the Declaration of Helsinki. All subjects received an explanation of the study and provided informed consent.

### 2.2 Questionnaire and medical history

All subjects completed a questionnaire with questions regarding age, sex, medical history and Barthel index.^17^

### 2.3 Physical assessment

Body composition was assessed by bioelectrical impedance analysis using the InBody S10 system (JMW140; Biospace Co., Ltd., Seoul, Korea), on the participant. Skeletal muscle mass index (SMI) was calculated as the mass of appendicular skeletal muscle divided by the square of the height.^18^ Body mass index (BMI) was calculated as weight divided by the square of the height.

### 2.4 Tongue muscle strength

Tongue pressure was measured using a tongue pressure measurement device (TPM‐01; JMS Co., Ltd), according to the method described by Tsuga et al^19^ The subject was asked to sit in a relaxed state, and to place a balloon on a plastic probe against the front of the palate. The subject was then required to raise the tongue and press the balloon against the palate as hard as possible for approximately 7 seconds. Tongue pressure was measured three times at 1‐min intervals, and maximum tongue pressure (MTP) was recorded.

### 2.5 Oral intake functional assessment

Oral intake function was examined by mealtime observation such as the diet level and respiratory pattern changes, and using videofluoroscopy. Oral intake function was assessed using the Functional Oral Intake Scale (FOIS),^20^ an ordinal scale yielding scores from 1 to 7 that was developed to record the functional level of oral intake.

### 2.6 Videofluoroscopic swallowing study

Videofluoroscopic swallowing study (VFSS) was carried out using an X‐ray fluoroscopic apparatus (VC‐1000; Hitachi Medical Corp.). Subjects had a 20.0‐mm metal rod attached to the chin region as a distance correction indicator and were seated in a dedicated videofluoroscopy chair (back at 90°). Profile images were taken with the subject in a natural head position. Imaging conditions were tube voltage, 65‐75 kV; and tube current, 1.25‐2.5 mA. VF imaging was carried out in automatic brightness control mode in which conditions were automatically changed according to the movement or position of the subject. The test food during the VFSS comprised 3.0 mL of moderately thick liquid containing barium sulphate,^21^, ^22^ taking into account the reduced risk of pulmonary aspiration as well as induction of the swallowing reflex. Each subject was first instructed to breathe deeply and then to slowly expel all air, and an image was recorded at rest. Next, the test food was introduced under the tongue of the subject using a syringe, and the subject was commanded to swallow. VF images were saved in AVI format at 30 frames/second, and stored on a personal computer. The laryngeal position (vertical position) was measured from the image taken at rest. Laryngeal anterior displacement, laryngeal superior displacement, and change in posterior pharyngeal wall advancement were analysed from the video recordings. Measurements of the laryngeal position and posterior pharyngeal wall advancement were made using ImageJ software (United States National Institutes of Health, Bethesda, MD).^23^ Laryngeal displacement was measured using 2‐dimensional motion analysis software (Dipp‐Motion V/ 2D; DITECT Corp.).

#### 2.6.1 Laryngeal position at rest (LPR)

**FIGURE 1:**
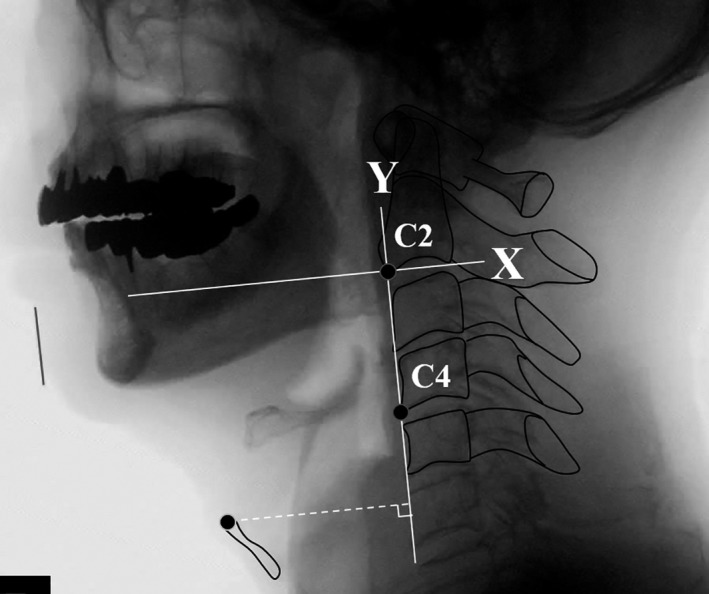
Lateral view from a videofluoroscopic swallowing study with marked points and lines for laryngeal position at rest. The straight line joining the anteroinferior corner of the second cervical vertebra (C2) and the anteroinferior corner of the fourth cervical vertebrae (C4) is taken as the y‐axis. The line perpendicular to the y‐axis passing through C2 is set as the x‐axis. Laryngeal position at rest (LPR) is measured by drawing a line perpendicular to the y‐axis from the anterosuperior margin of the thyroid cartilage, and measuring the distance from the point of intersection to the anteroinferior corner of C2

The straight line joining the anteroinferior corner of the second cervical vertebra (C2) and the anteroinferior corner of the fourth cervical vertebrae (C4) was taken as the y‐axis. The line passing through the anteroinferior corner of C2 perpendicular to the y‐axis was set as the x‐axis. LPR was measured by drawing a line perpendicular to the y‐axis from the anterosuperior margin of the thyroid cartilage,^24^ and measuring the distance from the point of intersection to the anteroinferior corner of C2 (Figure 1). LPR value was standardised using the distance between the anteroinferior corner of C2 and the anteroinferior corner of C4 to correct for body size differences, and expressed as an index.

#### 2.6.2 Laryngeal anterior displacement (LAD) and laryngeal superior displacement (LSD)

**FIGURE 2:**
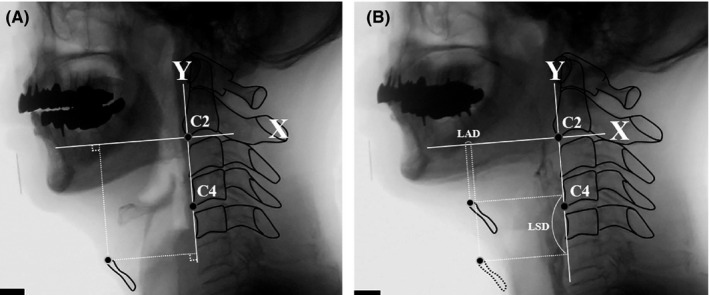
Lateral view from a videofluoroscopic swallowing study with marked points and lines for laryngeal anterior displacement (LAD) and laryngeal superior displacement (LSD). The straight line joining the anteroinferior corner of the second cervical vertebra (C2) and the anteroinferior corner of the fourth cervical vertebrae (C4) is taken as the y‐axis. The line perpendicular to the y‐axis passing through C2 is set as the x‐axis. LAD and LSD were determined by subtracting (a) the initial position of the thyroid cartilage from (b) peak position reached in the X direction and Y direction, respectively, during swallowing

The path of the anterosuperior margin of the thyroid cartilage was recorded. Laryngeal anterior and superior displacements were determined by subtracting the initial position of the thyroid cartilage from the peak position reached in the X direction and in the Y direction, respectively, during swallowing (Figure 2).^25^

#### 2.6.3 Posterior pharyngeal wall advancement (PPWA)

**FIGURE 3:**
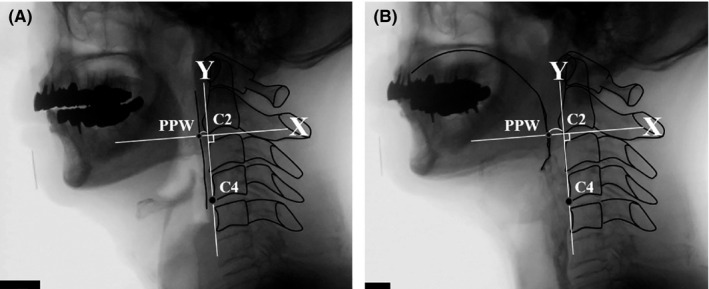
Lateral view from a videofluoroscopic swallowing study with marked points and lines for posterior pharyngeal wall advancement (PPWA). The straight line joining the anteroinferior corner of the second cervical vertebra (C2) and the anteroinferior corner of the fourth cervical vertebrae (C4) is taken as the y‐axis. The line perpendicular to the y‐axis passing through C2 is set as the x‐axis. The distance from an intersection point (PPW) of the posterior pharyngeal wall and the x‐axis to the origin was measured from images (a) at rest and (b) at maximum constriction

Distance from the intersection of the posterior pharyngeal wall and x‐axis (PPW) to the origin was measured from images at rest and at maximum constriction when swallowing the moderately thick liquid (Figure 3).^26^ PPWA was defined as the difference in distance between maximum constriction and rest.

### 2.7 Sample size

The effect size is 0.30 (moderate effect size). Using G*Power 3.1^27^ (Kiel University, Kiel, Germany), the smallest sample size was estimated as 82, with an alpha level of *P* = .05 and power of 0.80, using correlation analysis.

### 2.8 Statistical analysis

Correlations between parameters were evaluated using simple bivariate correlation. Parametric values were analysed using Pearson's product‐moment correlation coefficient, and non‐parametric values were analysed using Spearman's rank correlation coefficient. Multiple regression analysis was performed to assess relationships between SMI, MTP and PPWA.

Data were analysed using the Japanese version of SPSS for Windows version 26.0 (IBM Corp). All results are presented as mean ± standard deviation (SD). Values of *P* < .05 were considered statistically significant.

## 3 RESULTS

Mean age was 80.6 ± 6.8 years, mean Barthel index was 91.9 ± 14.8 (median, 100), mean SMI was 6.9 ± 0.8 kg/m^2^, and mean BMI was 21.4 ± 3.1 kg/m^2^. For FOIS, 14 subjects (17.1%) were level 4, 39 subjects (47.6%) were level 5, 27 subjects (32.9%) were level 6, and 2 subjects (2.4%) were level 7. Mean MTP was 23.5 ± 9.5 kPa. Mean distance measurements obtained from the VFSS were: LAD, 8.7 ± 3.5 mm; LSD, 35.9 ± 7.8 mm; and PPWA, 7.6 ± 2.1 mm. Mean LPR was 1.5 ± 0.3.

Table 1 shows correlation analyses of the individual parameters. MTP showed significant relationships with age (*r* = −.34, *P* < .01), SMI (r = 0.40, *P* < .01), and LSD (r = 0.22, *P* < .05). PPWA showed significant relationships with age (*r* = .22, *P* < .05), SMI (*r* = −.30, *P* < .01), and MTP (*r* = −.30, *P* < .01). LSD showed significant correlations with age (*r* = −0.28, *P* < .05) and SMI (*r* = 0.26, *P* < .05). Based on these results, multiple regression analysis was performed to assess relationships between MTP, PPWA and SMI, considering effects of age and LSD. Because LSD correlated significantly with age, SMI and MTP, LSD was also considered a confounder. MTP correlated significantly to SMI and PPWA (Table 2). PPWA correlated significantly with MTP, but showed no significant relationship with SMI (Table 3).

**TABLE 1:** Bivariate simple correlation analyses of each parameter

	PPWA	LSD	LAD	LPR	MTP	SMI	Age
Age	0.22^a^, *	−0.28*	0.16^a^	0.15	−0.34**	−0.43**	1
SMI	−0.30^a^, **	0.26*	−0.00^a^	−0.05	0.40**	1	
MTP	−0.30^a^, **	0.22*	−0.00^a^	−0.11	1		
LPR	0.02^a^	0.10	0.06^a^	1			
LAD	0.19^a^	0.21^a^	1				
LSD	−0.10^a^	1					
PPWA	1						

Abbreviations

LAD, laryngeal anterior displacement; LPR, laryngeal position at rest; LSD, laryngeal superior displacement; MTP, maximum tongue pressure; PPWA, posterior pharyngeal wall advancement; SMI, skeletal muscle mass index.

Correlations between parameters were evaluated using simple bivariate correlation. Parametric values were analysed using Pearson's product‐moment correlation coefficient, and non‐parametric values were analysed using Spearman's rank correlation coefficient.

^a^ Values for Spearman's rank correlation coefficient,

* *P* < .05;

** *P* < .01

**TABLE 2:** Result of multiple regression analysis

Model	Unstandardised coefficient		Standardised coefficient	*t* value	*p* value	95.0% Cl for B	
	B	Standard error	β			Lower limit	Upper limit
(Constant)	25.825	19.093		1.353	0.180	−12.194	63.845
Age	−0.199	0.159	−0.142	−1.254	0.214	−0.515	0.117
SMI	2.613	1.291	0.233	2.024	0.046	0.042	5.184
LSD	0.104	0.126	0.086	0.826	0.411	−0.147	0.355
PPWA	−1.064	0.487	−0.236	−2.186	0.032	−2.034	−0.095

Abbreviations

LSD, laryngeal superior displacement; MTP, maximum tongue pressure; PPWA, posterior pharyngeal wall advancement; SMI, skeletal muscle mass index.

Dependent variable: MTP.

**TABLE 3:** Result of multiple regression analysis

Model	Unstandardised coefficient		Standardised coefficient	*t* value	*p* value	95.0% Cl for B	
	B	Standard error	β			Lower limit	Upper limit
(Constant)	9.444	4.253		2.221	0.029	0.976	17.913
Age	0.039	0.036	0.125	1.077	0.285	−0.033	0.111
SMI	−0.543	0.294	−0.219	−1.844	0.069	−1.129	0.043
MTP	−0.055	0.025	−0.248	−2.186	0.032	−0.105	−0.005
LSD	0.003	0.029	0.011	0.104	0.917	−0.054	0.060

Abbreviations

LSD, laryngeal superior displacement; MTP, maximum tongue pressure; PPWA, posterior pharyngeal wall advancement; SMI, skeletal muscles mass index.

Dependent variable: PPWA.

## 4 DISCUSSION

In the present study, SMI was evaluated as an index of muscle mass of the whole body, tongue pressure was evaluated as an index of tongue muscle strength, and the state of pharyngeal organs involved in swallowing and the dynamics of the pharyngeal organs as a result of swallowing movements was evaluated using VFSS. We attempted to use this to clarify the effects of reduced tongue muscle strength seen in dysphagia patients on pharyngeal movements during swallowing.

Lee et al^7^ evaluated the swallowing process in the oral phase, expressed by factors such as lip closure, mastication function, bolus formation, and oral transit time, in stroke patients, and reported that loss of tongue pressure is related to oral‐phase dysphagia. While numerous reports have examined the relationship between tongue pressure and dysphagia,^3^, ^4^, ^6^, ^8^ few appear to have investigated relationships between tongue pressure and pharyngeal conditions that result in pharyngeal‐phase dysphagia or pharyngeal movements associated with swallowing movements.^9^, ^10^

In the present study, MTP showed a positive correlation with LSD. The tongue pressure generation is associated with the activation of the suprahyoid muscles as well as the tongue muscles. ^28^, ^29^ Sunada et al^30^ reported that both the tongue body and hyoid are attached to many muscles, including the suprahyoid and infrahyoid muscles that move the larynx, and that the tongue movement of pushing against the hard palate was effective for activating hyoid muscle activity and elevating the larynx. These may be one of the reasons that the tongue pressure and the amount of laryngeal elevation were related.

A decrease in MTP correlated with a decrease in SMI, supporting the results of Kobuchi et al^31^ PPWA was not associated with SMI. This result is at odds with the finding that MTP was significantly associated with SMI. Motor innervation of the tongue muscles, which are important for swallowing, occurs via the hypoglossal nerve. Embryologically, these muscles are striated muscle deriving from somites, and thus have the same origin as the somatic muscle that constitutes the skeletal muscle of the limbs. Tongue muscle strength is therefore conjectured to be affected by skeletal muscle mass. Pharyngeal muscle, on the other hand, is innervated by the glossopharyngeal and vagus nerves, and is striated muscle derived from the fourth branchial arch. Other than during swallowing, pharyngeal muscle is controlled by the respiratory centre and exhibits activity linked mainly to exhalation.^13^, ^32^ According to Elabie et al,^14^ immunohistochemical studies indicate that ageing‐related changes in pharyngeal muscles may differ from the loss of muscle mass and strength commonly seen in ageing‐related changes to skeletal muscle. Consequently, these histological features of pharyngeal muscle may be why the movements of the pharyngeal muscle may not be readily affected by skeletal muscle mass.

Posterior pharyngeal wall advancement showed a negative correlation with MTP. This means that the reduction in tongue muscle strength is related to increased PPWA. Reduced tongue function and reduced tongue pressure have been reported in tongue cancer patients following surgery.^33^ Hartl et al^16^ used cine‐MRI to investigate post‐surgery tongue cancer patients, and reported that increased PPWA was observed. Fujiu et al^15^ similarly observed increased PPWA, and considered this phenomenon to represent a compensatory action in the movements of swallowing. Furthermore, Hammer et al^34^ found that swallowing pressure was maintained even when posterior movement of the tongue was restricted by tongue holding, and attributed this to the medullary swallowing interneurons reacting to changes in tongue position, regulating movements of the superior pharyngeal constrictor muscle and the tongue. The increased PPWA seen in patients with reduced tongue pressure in the present study may be an expression of compensatory movements to maintain pharyngeal constrictor pressure. To the best of our knowledge, this is the first study to show a relationship between skeletal muscle mass and PPWA, which possibly results in swallowing pressure. In addition, the finding in this study that PPWA compensates for decreases in tongue pressure represents useful information when considering training plans for dysphagia rehabilitation.

A number of limitations to the present study need to be considered. The first is that all subjects were men. Sex differences in tongue muscle strength^35^ and skeletal muscle mass^18^ are known to be present in the elderly, and effects with ageing also differ.^36^, ^37^ Subjects in this study were thus typically elderly men. Results with women in the target population need to be determined.

The second limitation was that tongue muscle strength was assessed using voluntary MTP, and measurements were not made during functioning. However, tongue pressure measurement with a probe is widely used at present as a tool for quantitative assessment of tongue muscle strength and tongue function,^19^, ^33^ and a relationship with dysphagia has already been identified in numerous reports.^3^, ^4^, ^6^ Measurement of tongue pressure using a probe was thus adopted in this study.

A third limitation was that swallowing movements were only measured in two dimensions by VFSS, and no three‐dimensional evaluations were performed. Three‐dimensional analysis of the pharynx is possible using cine‐MRI,^16^ and this may reveal new knowledge. However, since swallowing was assessed in the present study using VFSS as the gold standard,^24^ the present results may be applicable to clinical diagnosis and could provide information that is useful for clinical practice in daily.

## 5 CONCLUSIONS

The results showed that posterior pharyngeal wall advancement during swallowing was not readily affected by skeletal muscle mass, and pointed to the possibility that increased posterior pharyngeal wall advancement may compensate for swallowing function in individuals with reduced tongue muscle strength.

## CONFLICT OF INTEREST

The authors declare no conflict of interest.

## AUTHOR'S CONTRIBUTION

Indicate authors’ role in study concept and design, acquisition of subjects and/or data, analysis and interpretation of data, and preparation of manuscript. Takeshi Kikutani conceived and designed the study, conducted the study, interpreted the data and prepared the manuscript. Keigo Nagashima acquired the subjects and data, performed statistical analysis, interpreted the data and prepared the manuscript. Taishi Miyashita acquired the subjects and data, interpreted the data and prepared the manuscript. Yuri Yajima and Fumiyo Tamura interpreted the data and prepared the manuscript.

### Peer Review

The peer review history for this article is available at https://publons.com/publon/10.1111/joor.13120.
